# Improved Cultivars and the Application of Combined Fertilizer Improve the Grain Yield and the Nitrogen Uptake and Utilization in Continuously Cropped Soybean (*Glycine max* (L.) Merr.)

**DOI:** 10.3390/plants15050845

**Published:** 2026-03-09

**Authors:** Wenbo Liu, Demin Rao, Futi Xie, Haiying Wang, Xingdong Yao

**Affiliations:** 1 Hebei Key Laboratory of Crop Stress Biology, College of Agronomy and Biotechnology, Hebei Normal University of Science & Technology, Qinhuangdao 066000, China; lwbsynydx@163.com; 2 Soybean Research Institute, Shenyang Agricultural University, Shenyang 110866, China; xft299@syau.edu.cn; 3 Soybean Research Institute, Jilin Academy of Agricultural Sciences, National Engineering Research Center of Soybean, Changchun 130033, China; rdm1397155464@163.com

**Keywords:** continuous cropping, soybean, cultivar renewal, nutrient application

## Abstract

In recent years, continuous cropping has become a major constraint on soybean production in China, and thus, researching methods to improve soybean yield under this cropping pattern has become a research hotspot. This study aimed to explore whether cultivar improvement and different fertilization regimes could enhance the nutrient uptake and resource utilization of continuously cropped soybean, thereby elevating its yield potential. A total of 11 soybean cultivars were subjected to different fertilization treatments, with the leaf area index (LAI), net photosynthetic rate (Pn), radiation use efficiency (RUE), dry matter accumulation, crop growth rate (CGR), nitrogen content (NC), grain yield, yield components, and harvest index (HI) analyzed. Compared with early cultivars, current cultivars increased LAI, Pn, CGR, RUE, NC, dry matter accumulation, grains per plant, 100-seed weight, HI, and grain yield by 26.22%, 10.07%, 34.13%, 22.65%, 20.43%, 29.44%, 30.09%, 9.80%, 13.69%, and 15.88%, respectively, while decreasing the nitrogen requirement per 100 g grain by 20.08%. Similarly, compared with unfertilized plants, fertilized plants increased these indices by 23.93%, 14.08%, 53.38%, 39.01%, 29.53%, 42.49%, 16.95%, 23.35%, 10.49%, and 26.50%, respectively, while decreasing the nitrogen requirement per 100 g grain by 14.40%, with the highest yield observed by the 2006-era cultivar (Dennison) fed compound fertilizer. In conclusion, cultivar improvement and fertilization can improve the yield potential of continuously cropped soybean by enhancing light energy and optimizing nitrogen accumulation and consumption, and future research should focus more on breeding to further tap the production potential of continuously cropped soybean.

## 1. Introduction

Soybean (*Glycine max* (L.) Merr.) is an important crop with a 5000-year cultivation history in China. As a globally important oilseed and protein crop, soybean plays a critical role in food security, livestock feed production, and the agricultural economy [1]. The world’s major soybean-producing countries include the United States, Brazil, Argentina, China, and India, which together dominate global soybean production and trade. Soybean is widely used as a high-quality protein source in animal feed, and provides edible oil, protein products, and raw materials for the food processing industry.

At present, the arable land available in Northeast China is limited, and continuous cropping has become prevalent due to the intensive cultivation of soybean [2]. In Heilongjiang Province alone, the soybean planting area reached 3.5812 million hectares [3], with 70% to 90% of this area under continuous cropping. Such practice causes the partial depletion of soil nutrients and alters soil physicochemical properties, which, in turn, induce continuous cropping obstacles [4] and a decline in grain yields [5]. Thus, addressing the challenges posed by continuous cropping in soybean production is an urgent priority [6].

With the advancement of breeding theories and methods [7,8], breeders have been continuously developing new soybean cultivars and upgrading existing ones. Cultivar improvement can effectively boost soybean grain yields. Thanks to such efforts, soybean production has increased by 16.8 kg ha^−1^ per year in the United States, 23 kg ha^−1^ per year in India, and 13 kg ha^−1^ per year in Canada [9]. In the present study, 11 soybean cultivars released from the 1960s to the 2000s were employed, namely, Amsoy, Williams 82, Liaodou 3, Resnik, Liaodou 10, Liaodou 11, Kottman, Liaodou 12, Tiefeng 31, Dilworth, and Dennison. Additionally, cultivar improvement has enhanced the net photosynthetic rate, stomatal conductance, nitrogen accumulation, crop growth rate, and radiation use efficiency of soybean plants [10,11,12,13], along with other agronomic traits. These improvements facilitate dry matter accumulation in soybean plants and thereby contribute to higher yields.

Cultivar improvement is the decisive factor in boosting soybean yields, while fertilizer application and cultivation techniques serve as essential guarantees for unlocking the yield potentials of soybean cultivars [14]. The increase in soybean grain yields relies heavily on fertilization, which is commonly adopted to replenish excessively depleted nutrient elements in continuous cropping systems, and thus, alleviates associated continuous cropping obstacles [15,16]. Furthermore, fertilization can effectively enhance the radiation use efficiency and dry matter accumulation [17,18], thereby increasing soybean grain yields.

Previous studies have mainly focused on cultivar improvement and fertilization practices for soybean under rotation systems, while continuous cropping often causes soil degradation, restricted nitrogen uptake, and yield reduction [19,20,21]. However, few studies have clarified the interactive effects of cultivar genetic improvement and fertilization on soybean performance under continuous cropping stress, and the related physiological mechanisms and genotype–environment interactions remain unclear. This study provides a new scientific contribution to understanding how cultivar improvement and combined fertilization improve photosynthetic performance, nitrogen accumulation, translocation, and grain yield in continuous cropping systems. To ensure the reliability and wide representativeness of our results, we selected 11 typical soybean cultivars released from 1966 to 2006 from similar latitude regions in the United States and China with comparable genetic backgrounds. The objectives were to (i) reveal the physiological mechanisms of cultivar improvement and fertilization that regulate light energy utilization, nitrogen accumulation, and yield formation under continuous cropping; (ii) clarify the genotype × continuous cropping stress × fertilization interaction on soybean productivity; and (iii) provide a theoretical basis for cultivar renewal and nutrient management in continuous cropping soybean production.

## 2. Results

### 2.1. Photosynthetic Characteristics Traits

#### 2.1.1. Net Photosynthetic Rate

Soybean growth, photosynthetic performance, dry matter production, and nitrogen accumulation are crucial physiological processes that determine the final grain yield. In this study, LAI, Pn, RUE, plant dry matter, and CGR were determined in the sixth node (V6), full bloom (R2), and full seed (R6) growth stages. Plant dry matter was also measured in the full maturity (R8) stage. In addition, nitrogen-related traits (plant NC, vegetative organ NC, grain NC, and nitrogen requirement per 100 g grain), along with yield and yield components (grain yield, grain number per plant, 100-grain weight, and HI), were determined to comprehensively evaluate the effects of cultivar improvement and combined fertilization on continuous cropping soybean.

**Figure 1:**
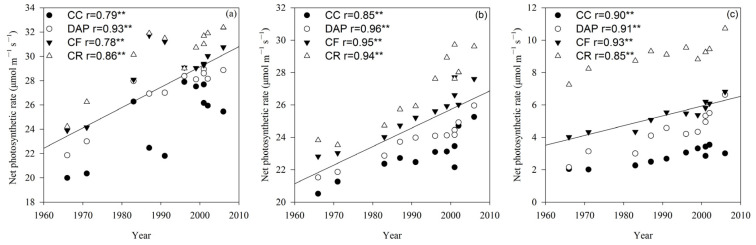
Net photosynthetic rate (Pn) of soybean cultivars under different fertilization treatments in the V6 (**a**), R2 (**b**) and R6 (**c**) growth stages. CC, continuous cropping; DAP, continuous cropping + diammonium phosphate fertilizer; CF, continuous cropping + compound fertilizer; CR, crop rotation. Slopes of regression lines: (**a**) CC 0.17, DAP 0.17, CF 0.15, and CR 0.17; (**b**) CC 0.09, DAP 0.09, CF 0.12, and CR 0.16; (**c**) CC 0.04, DAP 0.09, CF 0.06, and CR 0.06. **, significant at the 0.01 probability level. The lines represent the linear regression lines for each treatment. If all four treatments are significantly correlated, they are combined into one line.

Pn differed significantly (*p* < 0.05 or *p* < 0.01) between all the treatments in the V6 and R2 growth stages, whereas in the R6 stage, significant (*p* < 0.01) differences in Pn were observed between treatments, cultivars, and their interactions (Table 1). The Pn of soybean cultivars declined with advancing growth stages and increased significantly (*p* < 0.01) with the release year of cultivars (Figure 1). Under the continuous cropping (CC) treatment, Pn increased annually by 0.14 μmol m^−2^ s^−1^ at V6, 0.11 μmol m^−2^ s^−1^ at R1, and 0.02 μmol m^−2^ s^−1^ at R6.

#### 2.1.2. Leaf Area Index

**Figure 2:**
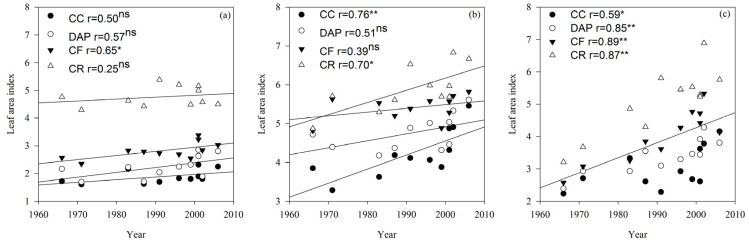
Leaf area index (LAI) of soybean cultivars under different fertilization treatments in the V6 (**a**), R2 (**b**) and R6 (**c**) growth stages. CC, continuous cropping; DAP, continuous cropping + diammonium phosphate fertilizer; CF, continuous cropping + compound fertilizer; CR, crop rotation. Slopes of regression lines: (**a**) CC 0.009, DAP 0.017, CF 0.015, and CR 0.007; (**b**) CC 0.036, DAP 0.018, CF 0.010, and CR 0.031; (**c**) CC 0.028, DAP 0.034, CF 0.055, and CR 0.068. *, significant at the 0.05 probability level; **, significant at the 0.01 probability level; ns, not significant at the 0.05 probability level. The lines represent the linear regression lines for each treatment. If all four treatments are significantly correlated, they are combined into one line.

The LAI in the V6, R2 and R6 growth stages differed significantly (*p* < 0.01) between all the treatments, cultivars, and their interactions (Table 1). The LAI of the soybean cultivars increased rapidly at first and then decreased with advancing growth stages, reaching the maximum value in the R2 growth stage. In the R6 growth stage, the LAI was significantly (*p* < 0.05 or *p* < 0.01) correlated with the cultivar release year under the CC, continuous cropping + diammonium phosphate fertilizer (DAP), continuous cropping + compound fertilizer (CF), and crop rotation (CR) treatments (Figure 2). Both LAI and Pn in all growth stages exhibited an increasing trend in the order of CR > CF > DAP > CC (Figure 1 and Figure 2).

### 2.2. Radiation Use Efficiency

**Figure 3:**
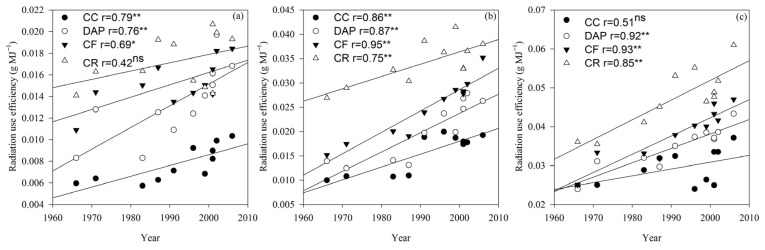
Radiation use efficiency (RUE) of soybean cultivars under different fertilization treatments in the V6 (**a**), R2 (**b**) and R6 (**c**) growth stages. CC, continuous cropping; DAP, continuous cropping + diammonium phosphate fertilizer; CF, continuous cropping + compound fertilizer; CR, crop rotation. Slopes of regression lines: (**a**) CC 0.0001, DAP 0.0002, CF 0.0001, and CR 0.0001; (**b**) CC 0.0003, DAP 0.0004, CF 0.0004, and CR 0.0003; (**c**) CC 0.0002, DAP 0.0004, CF 0.0005, and CR 0.0005. *, significant at the 0.05 probability level; **, significant at the 0.01 probability level; ns, not significant at the 0.05 probability level. The lines represent the linear regression lines for each treatment. If all four treatments are significantly correlated, they are combined into one line.

RUE differed significantly (*p* < 0.01) between the cultivars and treatments (Table 1). In the R2 and R6 growth stages, RUE was significantly (*p* < 0.05 or *p* < 0.01) correlated with the cultivar release year under the DAP and CF treatments. Under the CR treatment, RUE was significantly (*p* < 0.01) and positively correlated with the cultivar release year in the R2 and R6 growth stages. Under the CC treatment, RUE was significantly (*p* < 0.01) and positively correlated with the cultivar release year in the V6 and R2 growth stages, with annual increases of 0.0001 and 0.0002 g MJ^−1^, respectively (Figure 3).

### 2.3. Dry Matter Accumulation

**Figure 4:**
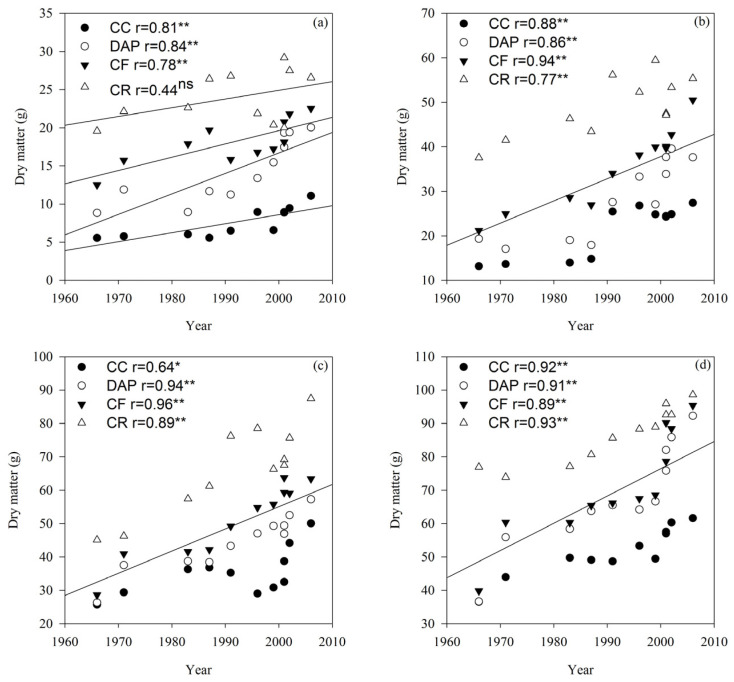
Dry matter weight per plant of soybean cultivars under different fertilization treatments in the V6 (**a**), R2 (**b**), R6 (**c**) and R8 (**d**) growth stages. CC, continuous cropping; DAP, continuous cropping + diammonium phosphate fertilizer; CF, continuous cropping + compound fertilizer; CR, crop rotation. Slopes of regression lines: (**a**) CC 0.12, DAP 0.27, CF 0.17, and CR 0.11; (**b**) CC 0.40, DAP 0.57, CF 0.63, and CR 0.40; (**c**) CC 0.35, DAP 0.61, CF 0.81, and CR 0.89; (**d**) CC 0.52, DAP 1.07, CF 1.09, and CR 0.59. *, significant at the 0.05 probability level; **, significant at the 0.01 probability level; ns, not significant at the 0.05 probability level. The lines represent the linear regression lines for each treatment. If all four treatments are significantly correlated, they are combined into one line.

The dry matter weight differed significantly (*p* < 0.01) between the cultivars and treatments in the V6, R2, and R6 growth stages (Table 1). In general, the effect of the cultivar release year on the dry matter weight became increasingly pronounced with advancing soybean growth. Except for the CR treatment in the V6 growth stage, the dry matter weight increased linearly with cultivar release year (*p* < 0.05 or *p* < 0.01). Furthermore, the dry matter weight in all growth stages followed the order of CR > CF > DAP > CC (Figure 4).

### 2.4. Crop Growth Rate

**Figure 5:**
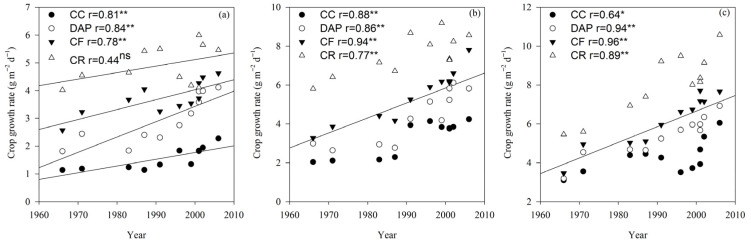
Crop growth rate (CGR) of soybean cultivars under different fertilization treatments in the V6 (**a**), R2 (**b**) and R6 (**c**) growth stages. CC, continuous cropping; DAP, continuous cropping + diammonium phosphate fertilizer; CF, continuous cropping + compound fertilizer; CR, crop rotation. Slopes of regression lines: (**a**) CC 0.02, DAP 0.06, CF 0.04, and CR 0.02; (**b**) CC 0.06, DAP 0.09, CF 0.10, and CR 0.06; (**c**) CC 0.04, DAP 0.07, CF 0.10, and CR 0.11. *, significant at the 0.05 probability level; **, significant at the 0.01 probability level; ns, not significant at the 0.05 probability level. The lines represent the linear regression lines for each treatment. If all four treatments are significantly correlated, they are combined into one line.

The CGR differed significantly (*p* < 0.01) between the cultivars and treatments (Table 1). In the V6, R2, and R6 growth stages, CGR was significantly (*p* < 0.01) and positively correlated with the cultivar release year under the DAP and CF treatments. Under the CR treatment, CGR was significantly (*p* < 0.01) and positively correlated with the cultivar release year in the R2 and R6 growth stages. In the V6, R2, and R6 growth stages, CGR was significantly (*p* < 0.05 or *p* < 0.01) correlated with the cultivar release year under the CC treatment, with annual increases of 0.03, 0.06, and 0.07 g m^−2^ d^−1^, respectively (Figure 5).

### 2.5. Nitrogen Accumulation

**Figure 6:**
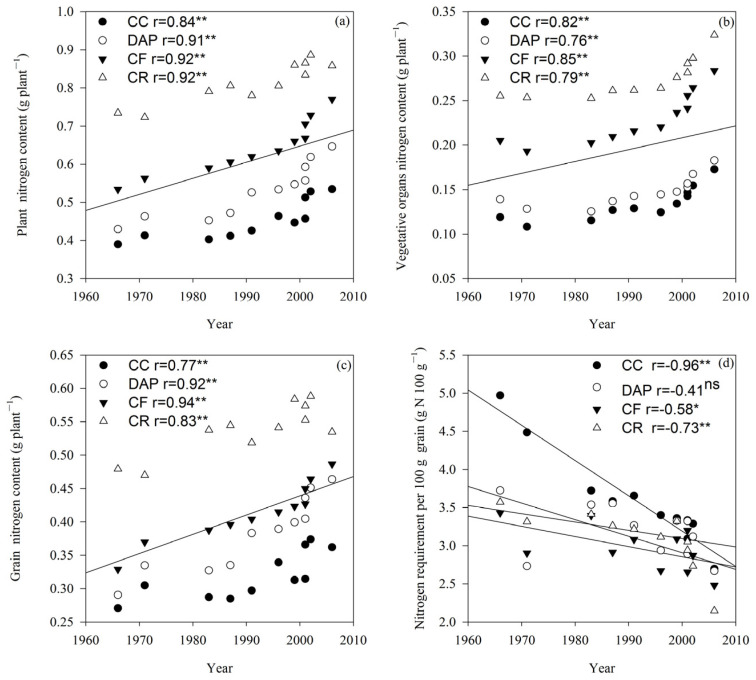
Soybean plant nitrogen content (NC) (**a**), vegetative organ NC (**b**), grain NC (**c**) and nitrogen requirement per 100 g grain (**d**) under different fertilization treatments. CC, continuous cropping; DAP, continuous cropping + diammonium phosphate fertilizer; CF, continuous cropping + compound fertilizer; CR, crop rotation. Slopes of regression lines: (**a**) CC 0.003, DAP 0.005, CF 0.005, and CR 0.004; (**b**) CC 0.001, DAP 0.001, CF 0.002, and CR 0.001; (**c**) CC 0.002, DAP 0.004, CF 0.003, and CR 0.002; (**d**) CC −0.046, DAP −0.011, CF −0.013, and CR −0.022. *, significant at the 0.05 probability level; **, significant at the 0.01 probability level; ns, not significant at the 0.05 probability level. The lines represent the linear regression lines for each treatment. If all four treatments are significantly correlated, they are combined into one line.

The plant NC, vegetative organ NC, and nitrogen requirement per 100 g of grain differed significantly (*p* < 0.01) between the cultivars and treatments, whereas the grain NC differed significantly (*p* < 0.05 or *p* < 0.01) between the cultivars, treatments, and their interactions (Table 1). Under all treatments, the plant NC, vegetative organ NC, and grain NC showed consistent responses to the cultivar release year, increasing linearly with advancing release year (*p* < 0.01, Figure 6a–c). In contrast, the nitrogen requirement per 100 g grain exhibited the opposite trend to that of the plant NC, vegetative organ NC, and grain NC. Overall, the nitrogen requirement per 100 g grain decreased linearly with the cultivar release year across all treatments (Figure 6d). The plant NC, vegetative organ NC, and grain NC in all growth stages followed the order of CR > CF > DAP > CC (Figure 6a–c).

### 2.6. Grain Yield and Yield Components

**Figure 7:**
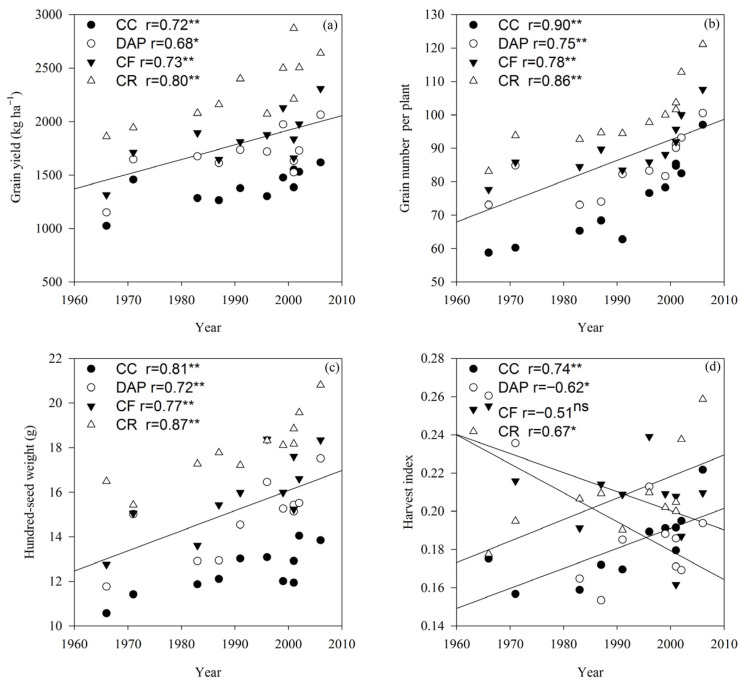
Soybean grain yield (**a**), grain number per plant (**b**), hundred-seed weight (**c**) and harvest index (HI) (**d**) under different fertilization treatments. CC, continuous cropping; DAP, continuous cropping + diammonium phosphate fertilizer; CF, continuous cropping + compound fertilizer; CR: Crop rotation. Slopes of regression lines: (**a**) CC 9.13, DAP 12.11, CF 14.49, and CR 19.03; (**b**) CC 0.07, DAP 0.09, CF 0.11, and CR 0.10; (**c**) CC 0.84, DAP 0.48, CF 0.49, and CR 0.65; (**d**) CC 0.001, DAP −0.002, CF −0.001, and CR 0.001. *, significant at the 0.05 probability level; **, significant at the 0.01 probability level; ns, not significant at the 0.05 probability level. The lines represent the linear regression lines for each treatment. If all four treatments are significantly correlated, they are combined into one line.

The grain yield differed significantly (*p* < 0.01) between the cultivars, treatments, and their interactions (Table 1). The grain yield increased linearly with the cultivar release year (*p* < 0.05 or *p* < 0.01), with an annual increase rate of 14.79 kg ha^−1^ under the CC treatment. Under the CC treatment, the highest grain yield of soybean cultivars reached 1617 kg ha^−1^, which was 57.70% higher than the lowest yield. Relative to the grain yield under the CC treatment, the annual proportional increases under the DAP, CF, and CR treatments were 0.52%, 0.80%, and 1.63%, respectively (Figure 7a).

The grain number per plant differed significantly (*p* < 0.01) between the cultivars, treatments, and their interactions, whereas the hundred-seed weight differed significantly (*p* < 0.01) between the cultivars and treatments (Table 2). The grain number per plant and hundred-seed weight showed the same trends as the grain yield, increasing linearly with the cultivar release year (*p* < 0.01), with a marked increase in grain number per plant observed under the CR treatment. The HI increased linearly with the cultivar release year only under the CC and CR treatments (*p* < 0.05 or *p* < 0.01, Figure 7b–d).

## 3. Discussion

The decline in arable land and the rising demand for soybean have led to the widespread practice of continuous soybean cropping in Northeast China. Continuous cropping affects the growth and development of soybean plants, consequently reducing grain yields [5,22]. Thus, improving the soybean grain yield under continuous cropping conditions has become a research hotspot.

Cultivar improvement and rational fertilization are well recognized as effective measures for increasing soybean grain yields [23,24]. This study’s results indicate that under continuous cropping conditions, the grain yields of modern cultivars were higher than those of older ones, with an annual increase of 14.79 kg ha^−1^. This finding demonstrates that cultivar improvement enhanced the yield potential of soybean under continuous cropping, which was consistent with conclusions reported for crop rotation systems [23]. Previous studies have confirmed that genetic yield gains in soybean are achieved by improving the biomass accumulation and harvest index [25,26,27]. This study’s results further show that the soybean harvest index increased progressively with the cultivar release year under continuous cropping. Dry matter accumulation is the fundamental basis for soybean grain yield formation, and cultivar improvement has been shown to promote crop dry matter accumulation, thereby elevating grain yields [28]. In this study, the individual plant dry matter weight of soybean cultivars increased with the release year under continuous cropping, showing a significant, positive correlation. These results indicate that the increase in soybean grain yield under continuous cropping was driven by the improvement in biomass. Fertilization plays a crucial role in sustaining high grain yields in crop production [29]. The present study found that fertilization significantly increased the soybean grain yield and dry matter weight; the yield increase rates under the CF (20.90%) and DAP (32.11%) treatments were higher than those under the CC treatment. This suggests that multiple soil nutrients are partially depleted under continuous cropping, and replenishing these nutrients through fertilization can effectively mitigate continuous cropping obstacles [30], with compound fertilizer application being more conducive to improving grain yields in continuously cropped soybean. However, the grain yields and dry matter accumulation under crop rotation were higher than those under all fertilization treatments. This indicates that soybean is susceptible to continuous cropping stress, and fertilization can alleviate such obstacles and enhance the yield potential of soybean cultivars, but it could not achieve the yield effect of crop rotation [31]. Collectively, the results suggest that selecting current soybean cultivars combined with compound fertilizer application can better alleviate continuous cropping obstacles and effectively increase grain yields in continuously cropped soybean production.

Soybean grain yield is closely associated with the leaf photosynthetic rate and light energy interception [32,33,34]. Continuous soybean cropping can reduce the leaf photosynthetic rate and grain yield, with both showing a decreasing trend as the duration of continuous cropping increases. Previous studies have shown that the net photosynthetic rate of new soybean cultivars was 9.5% and 17.3% higher in the R2 and R4 growth stages under crop rotation, respectively [35]. In the present study, the trends of the net photosynthetic rate and leaf area index in the continuously cropped soybean were consistent with this finding, as both indices increased with the cultivar release year.

The radiation use efficiency is defined as the ability to convert absorbed photosynthetically active radiation into biomass [36] and reflects the matter production capacity of the crop [37]. Enhancing the radiation use efficiency can effectively boost crop yields [17]. Tollenaar and Aguilera [13] reported a 33% difference in the crop growth rate between older and newer cultivars grown at their respective optimal plant densities, with approximately 80% of this variation attributable to the higher radiation use efficiency of new cultivars. In this study, the radiation use efficiency and crop growth rate of continuously cropped soybean increased with the cultivar release year, demonstrating that cultivar improvement effectively elevated the net photosynthetic rate, radiation use efficiency, and crop growth rate, thereby increasing the soybean grain yield under continuous cropping conditions. Fertilization can maintain a higher net photosynthetic rate and leaf area index, improve the light energy utilization efficiency during the middle and late crop growth stages, and delay leaf senescence [38]. The results of this study indicate that the CF and DAP treatments significantly increased the net photosynthetic rate, leaf area index, radiation use efficiency, and crop growth rate in continuously cropped soybean, particularly during the late growth stage. This suggests that fertilization can enhance the light energy utilization efficiency and yield potential in continuously cropped soybean. In summary, selecting current soybean cultivars can improve the light energy utilization efficiency, fertilization can sustain such efficiency during the late growth and development stages of continuously cropped soybean, and the combination of cultivar improvement and fertilization enables continuously cropped soybean to effectively maintain high light energy utilization efficiencies [21].

Continuous soybean cropping can reduce plant nutrient levels, with a particularly extremely significant decline in plant nitrogen content [39], a trend that was also observed in the present study. Previous studies have shown that total nitrogen accumulation increases linearly with the cultivar release year in maize [40], barley [41], and wheat [42]. In this study, plant nitrogen content, vegetative organ nitrogen content, and grain nitrogen content of soybean all increased with the cultivar release year under continuous cropping, whereas the nitrogen requirement per 100 g of grain exhibited the opposite trend. This indicates that cultivar improvement can enhance the nitrogen uptake in continuously cropped soybean. Fertilization can promote the nitrogen accumulation, increase grain number per plant, and improve grain yield and quality in soybean [43]. In the present study, the plant nitrogen content, vegetative organ nitrogen content, and grain nitrogen content were all higher in fertilized soybean than in unfertilized plants under continuous cropping, while the nitrogen requirement per 100 g of grain showed the opposite trend. This implies that increased fertilization can achieve greater nitrogen accumulation in continuously cropped soybean. In summary, these results demonstrate that cultivar improvement and fertilization can improve the nitrogen accumulation and consumption in soybean under continuous cropping conditions.

## 4. Materials and Methods

### 4.1. Experimental Design

In 2014, field experiments that involved crop rotation (corn–corn–soybean rotation) and continuous cropping (continuous soybean monocropping) were initiated at the Liaozhong Long-term Positioning Test Base (41°52′ N, 123°12′ E, elevation 5.5–23.5 m) of Shenyang Agricultural University in Liaoning Province. The study area has a warm temperate subhumid continental climate, with a mean annual precipitation of 640 mm, a mean annual temperature of 8 °C, and a mean annual sunshine duration of 2527 h. The total experimental area was 4700 m^2^. The soil properties were as follows: total nitrogen, 1.26 g kg^−1^; soil organic matter, 11.15 g kg^−1^; available phosphorus, 37.30 mg kg^−1^; and available potassium, 161.78 mg kg^−1^.

**Figure 8:**
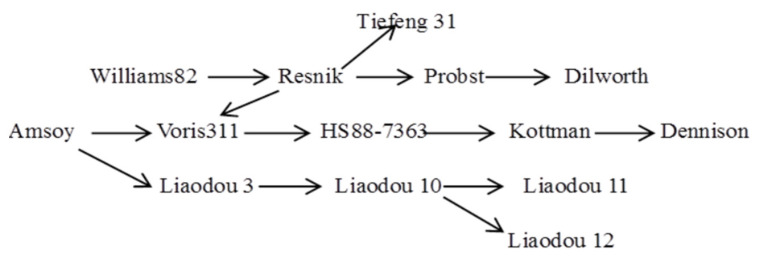
Genetic relationships of soybean cultivars.

This experiment was carried out during the 2019 and 2020 growing seasons, which were the sixth and seventh years of the long-term fixed-position trial. To ensure annual acquisition of soybean samples, the crop rotation experimental field was evenly divided into three sub-plots, corresponding to the corn–corn–soybean, corn–soybean–corn, and soybean–corn–corn rotations. A two-factor split-plot design was adopted, with different planting systems [continuous cropping (CC), crop rotation (CR), continuous cropping + diammonium phosphate fertilizer (DAP, pure N: 277.2 kg ha^−1^), and continuous cropping + compound fertilizer (CF, pure N: 90 kg ha^−1^; pure phosphorus: 90 kg ha^−1^; pure potassium: 90 kg ha^−1^)] as the main plots and different soybean cultivar treatments (the main soybean cultivars (Table 2), which were developed in different periods in Ohio, USA (38.45–41.22° N), and Liaoning Province, China (38.55–42.32° N), had a common ancestral parent; the genetic relationship of the soybean cultivars are shown in Figure 8) as the sub-plots. The difference in nitrogen rates between the DAP and CF treatments reflects the standard application rates for each fertilizer type, which were determined based on local typical fertilization practices when the long-term trial was established. The experiment was set with 3 replications and a total of 156 plots, each with 5 rows and a planting density of 150,000 plants ha^−1^. Prior to sowing, seeds were carefully selected, with 4 seeds sown per hole at a sowing depth of 3 cm. Thinning was performed in the seedling stage, retaining 2 healthy seedlings per hole. The experiment was conducted under rain-fed conditions, all fertilizers were applied once when sowing, and conventional field management practices were implemented throughout the growing period.

### 4.2. Determination of Items and Methods

#### 4.2.1. Dry Matter and Crop Growth Rate

Three plants with uniform growth were randomly selected in the sixth node (V6), full bloom (R2), full seed (R6), and full maturity (R8) growth stages of the soybean. Following organ separation, the plant tissues were first dried at 105 °C for 30 min, then oven-dried at 80 °C to a constant weight. The aboveground dry matter was weighed, and the average value was calculated as the dry matter weight of a single plant. The calculation formulas for dry matter weight per unit area and CGR were as follows [34]:

DMW = WPDMW × PD;

CGR = DMW/DAY;

where DMW is the dry matter weight per unit area (g m^−2^), WPDMW is the dry matter weight of a single plant (g plant^−1^), PD is plant density per unit area (plant m^−2^), CGR is the crop growth rate (g m^−2^ d^−1^), and DAY is the length of the soybean growing stage (days).

#### 4.2.2. Net Photosynthetic Rate and Radiation Use Efficiency

In the V6, R2, and R6 growth stages, three representative plants with uniform growth were selected for measurements. The net photosynthetic rate (Pn) of fully expanded leaves was measured using a portable photosynthesis system (LI-6400, Lincoln, NE, USA) between 9:00 and 11:00 on clear, sunny days [44,45]. Meanwhile, canopy photosynthetically active radiation (PAR) was determined with a canopy analyzer (AccuPAR LP-80, Pullman, WA, USA) from 11:00 to 13:00 on the same days. The measured canopy data were then used to calculate the leaf area index (LAI) based on the Lambert–Beer law, which assumes a random distribution of foliage and homogeneous canopy radiation attenuation [46], and the radiation use efficiency (RUE) of soybean was subsequently computed [47,48]:

Transmittance = I_t_/I_0_;

Extinction coefficient (K) = (−1/LAI) × ln (I_t_/I_0_);

IPAR = Qa × [1 − exp (−K × LAI)];

RUE = DMW/IPAR;

where I_t_ is the PAR in the lower layer (mol m^−2^ s^−1^), I_0_ is the PAR in the upper layer (mol m^−2^ s^−1^), LAI is the leaf area index, IPAR is the PAR interception of the canopy (MJ m^2^), Qa is the total incident solar radiation accumulated between two consecutive sampling dates (MJ m^−2^), K is the canopy light extinction coefficient, RUE is the radiation use efficiency (g MJ^−1^), and DMW is the dry matter weight per unit area (g m^−2^).

#### 4.2.3. Grain Yield and Harvest Index

In the R8 stage, three 3 m long rows of soybean plants were harvested from each plot to determine the grain yield. The water content of the grain yield was calculated as 13%. Additionally, 100-grain samples were randomly selected (*n* = 10 replicates) to measure the average hundred-seed weight. The harvest index (HI) was calculated [34] according to the formula below:

HI = (GY/DMW) × 100;

where HI is the harvest index (%), GY is the grain yield per unit area (kg ha^−1^), and DMW is the dry matter weight per unit area in the R8 growth stage (kg ha^−1^).

#### 4.2.4. Nitrogen Content and Nitrogen Requirement per 100 g Grain

The nitrogen content (NC) was determined using an automated Kjeldahl apparatus (KJELTEC™ 8400; FOSS Analytical, Hillerød, Denmark). The nitrogen requirement per 100 g grain was calculated as follows:

Nitrogen requirement per 100 g grain (g N 100 g^−1^) = (Grain NC/Grain weight) × 100.

### 4.3. Statistical Analysis

All measured data were processed using Microsoft Excel 2010. Statistical analyses were performed with SPSS software (Version 22.0, SPSS Inc., Chicago, IL, USA), and graphs were generated with Sigmaplot (Version 12, Systat Software).

In the analysis of variance (ANOVA) model appropriate for a split-plot design, planting system treatments were treated as the main-plot factor, soybean cultivar treatments as the sub-plot factor, and replication as a random factor. Planting system treatment effects were tested against the main-plot error, while cultivar and interaction effects were tested against the sub-plot error. Year was initially included as a fixed effect to test year-related interactions. No significant differences were observed between the two experimental years, and all year × treatment interactions were non-significant (*p* > 0.05) for all measured traits; therefore, data averaged across the two years were used for subsequent analyses. Mean values were compared using ANOVA followed by the least significant difference (LSD) test at the 0.05 level. Correlation coefficients were calculated using the Pearson method. A full combined ANOVA table for the grain yield across years, including year, year-related interactions, degrees of freedom, F-values, and *p*-values, is provided in the Supplementary Material (Table S1). The other traits were analyzed using the same model and showed similar patterns.

## 5. Conclusions

The results indicate that cultivar improvement and fertilization could enhance the photosynthetic performance, nitrogen uptake capacity, light energy use efficiency, and yield of continuously cropped soybean. The combination of new cultivars and compound fertilizer led to the most evident improvement in the net photosynthetic rate, nitrogen accumulation, and grain yield among all the continuous cropping treatments. This integrated strategy was more favorable for enhancing the resource use efficiency and grain yield in soybean, thus representing an effective approach to alleviating obstacles associated with continuous cropping. Nevertheless, no fertilization treatment could match the effect of crop rotation on yield, and only this practice could enable the full utilization of resources and tap the complete yield potential of soybean. This suggests that crop rotation remains the most effective agronomic strategy to break continuous cropping barriers and maximize productivity. Therefore, in actual production, it is recommended to select new cultivars and apply compound fertilizer to improve the resource use efficiency and yield of continuously cropped soybean. For regions with suitable conditions, the integration of reasonable rotation, improved cultivars, and optimized fertilization is suggested to achieve high-efficiency and high-yield soybean production.

## Figures and Tables

**Table 1:** Analysis of variance (ANOVA) results for soybean leaf area index, net photosynthetic rate, radiation use efficiency, dry matter, crop growth rate, nitrogen content, grain yield, yield components, and harvest index.

Trait	Treatment	Cultivar	Treatment × Cultivar
Leaf area index in V6 growth stage	**	**	**
Leaf area index in R2 growth stage	**	**	**
Leaf area index in R6 growth stage	**	**	**
Net photosynthetic rate in V6 growth stage	**	ns	ns
Net photosynthetic rate in R2 growth stage	*	ns	ns
Net photosynthetic rate in R6 growth stage	**	**	ns
Radiation use efficiency in V6 growth stage	**	**	ns
Radiation use efficiency in R2 growth stage	**	**	ns
Radiation use efficiency in R6 growth stage	**	**	ns
Plant dry matter in V6 growth stage	**	**	ns
Plant dry matter in R2 growth stage	**	**	ns
Plant dry matter in R6 growth stage	**	**	ns
Plant dry matter in R8 growth stage	**	**	ns
Crop growth rate in V6 growth stage	**	**	ns
Crop growth rate in R2 growth stage	**	**	ns
Crop growth rate in R6 growth stage	**	**	ns
Plant nitrogen content	**	**	ns
Vegetative organ nitrogen content	**	**	ns
Grain nitrogen content	**	**	*
Nitrogen requirement per 100 g grain	**	**	ns
Grain yield	**	**	**
Grain number	**	**	**
100-grain weight	**	**	ns
Harvest index	ns	ns	ns

Note: The asterisks indicate significance at the 0.05 (*) and 0.01 (**) levels of probability. “ns” indicates no significance.

**Table 2:** Tested soybean cultivars with similar growth maturity.

Cultivar	Year of Release	Institute of Seed Origin
Amsoy	1966	The Ohio State University
Williams 82	1971	The Ohio State University
Liaodou 3	1983	Liaoning Academy of Agricultural Sciences
Resnik	1987	The Ohio State University
Liaodou 10	1991	Liaoning Academy of Agricultural Sciences
Liaodou 11	1996	Liaoning Academy of Agricultural Sciences
Kottman	1999	The Ohio State University
Liaodou 12	2001	Liaoning Academy of Agricultural Sciences
Tiefeng 31	2001	Tieling Academy of Agricultural Sciences
Dilworth	2002	The Ohio State University
Dennison	2006	The Ohio State University

## Data Availability

The original contributions presented in this study are included in the article/Supplementary Materials. Further inquiries can be directed to the corresponding authors.

## Supplementary Materials

The following supporting information can be downloaded at https://www.mdpi.com/article/10.3390/plants15050845/s1, Table S1: Analysis of variance (ANOVA) for soybean grain yield across two years (2019–2020) under different treatments and cultivars.
