# Supplementary material for: Improved Cultivars and the Application of Combined Fertilizer Improve the Grain Yield and the Nitrogen Uptake and Utilization in Continuously Cropped Soybean (Glycine max (L.) Merr.)
Source: Plants (Basel). 2026 Mar 9;15(5):845. doi: 10.3390/plants15050845 (PMC12986734; doi:10.3390/plants15050845)
Supplement: Supplementary file 1 [file plants-15-00845-s001.zip › plants-4164693-supplementary.pdf]

Supplementary Table S1. Analysis of variance (ANOVA) for soybean grain yield across two years (2019–2020) under different treatments and cultivars.

Source of Variation	SS	DF	MS	F-Value	P-Value
Block	14340.5145	2			
Year (Y)	61.443	1	61.443	0.1313	0.7225
Treatment (T)	28343023.2926	3	9447674.4309	20193.9588	0.0001
Y × T	3650.7761	3	1216.9254	2.6011	0.0933
Main-plot error	6549.852	14	467.8466		
Cultivar (C)	11669481.0946	10	1166948.1095	1420.5693	0.0001
Y × C	6225.3931	10	622.5393	0.7578	0.669
T × C	3330463.647	30	111015.4549	135.1432	0.0001
Y × T × C	27502.7601	30	916.7587	1.116	0.3235
Sub-plot error	131434.4172	160	821.4651		
Total	43532733.1906	263			
